# Long Non-Coding RNA LINC01929 Facilitates Cell Proliferation and Metastasis as a Competing Endogenous RNA Against MicroRNA miR-1179 in Non-Small Cell Lung Carcinoma

**DOI:** 10.3389/bjbs.2022.10598

**Published:** 2022-07-14

**Authors:** Tinghong Pan, Hui Wang, Shuai Wang, Feng Liu

**Affiliations:** ^1^ Department of Thoracic Surgery, Yidu Central Hospital of Weifang, Weifang, China; ^2^ Department of Cardiothoracic Surgery, Zhucheng People’s Hospital, Weifang, China

**Keywords:** biomarker, prognosis, LINC01929, non-small cell lung carcinoma, miR-1179

## Abstract

**Introduction:** Non-small cell lung carcinoma (NSCLC) constitutes most lung cancers and has a poor prognosis. LncRNAs are a potential repository for the discovery of cancer prognostic markers. This study explored the role of LINC01929 in NSCLC, both the clinical prognostic significance and the mechanism of its influence on cells.

**Materials and Methods:** LINC01929 levels in 143 pairs of NSCLC tissues and non-cancerous tissues were detected by RT-qPCR. Kaplan-Meier curves and multivariate Cox regression assays were generated for evaluating the prognostic values of LINC01929. To evaluate the cellular function, an XTT assay and transwell invasion assays were performed.

**Results:** LINC01929 was up-regulated in NSCLC tissues compared with healthy tissues. A positive correlation was observed between LINC01929 expression level and tumor T (*p* = 0.002) or N stage (*p* = 0.010). Patients with higher LINC01929 levels had shorter overall survival (*p* = 0.009). Compared with other factors, high LINC01929 expression was significantly associated with poor survival in univariate Cox analysis (HR: 2.485, 95%CI: 1.220–5.060, *p* = 0.012). After multivariate Cox regression assays, LINC01929 was a independent prognostic factor (HR: 3.021, 95%CI: 1.377–6.628, *p* = 0.006). miR-1179 was a target miRNA of LINC01929. Inhibited expression of LINC01929 significantly reduced the proliferation, migration, and invasion of NSCLC cells by targeting miR-1179.

**Discussion:** This study revealed the upregulation of LINC01929 in NSCLC. This study supports previous studies showing LINC01929 as a potential prognostic factor for NSCLC.

## Introduction

The incidence rate and mortality rate of lung cancer remain high among various types of cancers [1]. In particular, lung cancer is ranked the most frequent cancer and causes the most cancer-related death in men, and ranked third and second in the incidence rate and mortality rate respectively in women [2, 3]. The mortality rate of lung cancer in China has been increasing over recent years [4]. Histologically, 85% of lung cancer cases are comprised of non-small cell lung carcinoma (NSCLC) [5]. The standard treatment for early-stage NSCLC is surgical lobectomy combined with lobe-specific or systematic lymph node dissection, and the 5-year overall survival is 48%–65% [6]. However, the five-year overall survival of stage IV NSCLC patients is only about 20% [7]. Given the differences in survival amongst NSCLC patients, more effective and reliable biomarkers are needed to determine prognosis and monitor response to therapies.

Tumor heterogeneity has been described in lung cancer and has an important impact on defining prognosis and therapy decisions [8]. Molecular heterogeneity is associated with mechanisms of lung cancer origin including genetic and epigenetic sources [9]. Recently, several studies have revealed the potential predictive roles of epigenetic expression modifications in lung cancer, particularly long noncoding RNAs (lncRNAs) [10, 11]. LncRNAs play crucial roles in many biological processes and are involved in the regulation of molecular signaling pathways resulting in changes in gene expression [12]. lncRNAs have been implicated in lung cancer progression and have the potential to act as biomarkers of prognosis [13]. For example, HOTAIR, with an increased expression in NSCLC, could be useful in the diagnosis and prognosis of lung cancer [14]. Recent findings showed a role of lncRNAs signatures for predicting the prognosis of lung adenocarcinoma, particularly lncRNAs AC124804.1 and MIR34AHG [15]. A meta-analysis study suggested and verified that high LINC00511 expression level correlated with poor prognosis in malignant tumors, including lung cancer [16].

LINC01929 has been reported to be dysregulated in NSCLC [15]. Therefore, it was hypothesized that aberrantly-expressed LINC01929 may serve as a useful prognostic marker in NSCLC. Hence, in the present study the expression levels of LINC01929 in NSCLC tissues and adjacent healthy tissue were investigated and related to clinical parameters. In addition, the effects of LINC01929 knockdown on its target miRNA expression in lung cancer cell lines were investigated.

## Materials and Methods

### Tissues Sources

Paired NSCLC tissues and non-cancerous tissue (NCT) from 143 participants were recruited at the Yidu Central Hospital of Weifang (Weifang, China) during the period between February 2011 and December 2015. Patients who had other cancers or had already received anti-tumor therapies (chemotherapy, radiation, or immunotherapy) were excluded. The medical records of these participants were reviewed, and the key parameters included are detailed in Table 1. Staging was carried out according to the IASLC 2009 classification system(International Association for the Study of Lung Cancer) [17]. Data on overall survival time from a 5-year follow-up survey were extracted and used in this analysis. All specimens and medical records were used by authorization with the submitted written informed consent from all participants. This study was approved by the institutional ethical board of Yidu Central Hospital of Weifang (2011–07).

**TABLE 1:** Correlation of the LINC01929 expression with clinical characteristics in NSCLC patients.

Study variables	Cases (*n* = 143)	LINC01929 level		*p*
		Low (*n* = 65)	High (*n* = 78)	
Age				0.151
≤60	72	37	35	
> 60	71	28	43	
Gender				0.926
Female	82	37	45	
Male	61	28	33	
Tumor size				0.086
≤ 3 cm	79	41	38	
> 3 cm	64	24	40	
Smoking status				
Non-smoker	74	33	41	0.831
Smoker	69	32	37	
Pathological type				
LUSC	58	21	37	0.067
LUAD/other	85	44	41	
Clinical T stage				
T1-T2	100	54	46	0.002
T3	43	11	32	
Clinical N stage				0.010
N0	94	50	44	
N1-N2	49	15	34	
Differentiation				0.398
Well/Moderate	87	42	45	
Poor	56	23	33	

Note: LUSC, lung squamous cell carcinoma; LUAD, lung adenocarcinoma.

### Cell Culture

Human NSCLC cell lines HCC-827 (CRL-2868), NCI-H522 (CRL-5810), NCI-H1703 (CRL-5889), and SK-MES-1 (HTB-58), were obtained from American Type Culture Collection (ATCC; Manassas, VA, United States) and grown in RPMI-1640 medium (HyClone, United States) with 10% fetal bovine serum (FBS; Gibco, United States) (v/v: 90/10). Normal human bronchial epithelial cell line BEAS-2B from ATCC (CRL-9609) was cultured in the same medium formulation (90% RPMI-1640 medium with 10% fetal bovine serum). The incubator condition of all cells was 5% CO_2_ in an air atmosphere at 37°C.

### Plasmids, siRNA, Inhibitors, and Transfection

Oligonucleotides for miR-1179 inhibition (anti-miR-1179), the specific siRNA for LINC01929 (si-LINC01929), and their corresponding negative controls (anti-NC and si-NC) were generated and purchased from GenePharma (Shanghai, China). A portion of the LINC01929 3′-UTR, containing miR-1179 predicted binding site (wild type, named as WT-LINC01929), and the 3′-UTR mutant with the miR-1179 binding sites mutated (MUT-LINC01929) was synthesized, verified, and cloned into luciferase reporter plasmids by Hanbio Biotechnolog (Shanghai, China) for the subsequent luciferase reporter assay. Oligonucleotides, siRNAs, or plasmids were subjected to transfection using Lipofectamine 3000 (Invitrogen, United States).

### RNA Isolation and Real-Time Quantitative PCR Assay

Frozen samples of human lung tissues were homogenized in liquid nitrogen. RNA was isolated from frozen human lung tissues and cultured cells using RNAiso Plus (Takara, China) and isopropyl alcohol (Aladdin, United States), followed by DNase treatment with Recombinant DNase I (RNase-free) (Takara, China). RNA concentration and purity were determined on a NanoDrop 1000 spectrophotometer (Thermo Fisher Scientific, United States). Total RNA (1 μg) was reverse transcribed using QuantiTect Reverse Transcription Kit (Qiagen, UK) according to the manufacturer’s instructions. RT-qPCR analysis was performed using SuperScript™ IV One-Step RT-PCR System at a StepOnePlus instrument (Applied Biosystems, United States). Primers were: 5′-CTT​GAC​ACG​ACT​TCA​GAA​GCC​TC-3' (forward) and 5′-GCA​GAG​CTC​GAC​CAG​GAC​AG-3' (reverse) for LINC01929; 5′-AGC​ATT​CTT​TCA​TTG​GTT​G-3' (forward) and 5′-GAA​CAT​GTC​TGC​GTA​TCT​C-3' (reverse) for miR-1179; 5′-GAA​GGT​GAA​GGT​CGG​AGT​C-3' (forward) and reverse 5′-GAAGATGGTGA TGGGATTTC-3′ for GAPDH; 5′-CGC​TTC​GGC​AGC​ACA​TAT​ACT​A-3' (forward) and 5′-CGC​TTC​ACG​AAT​TTG​CGT​GTC​A-3' (reverse) for U6. For LINC01929, the thermal profile was as follows: 95°C for 15 min followed by 50 cycles of 94°C for 15 s, Ta for 20 s and 60°C for 45 s. The cycling conditions for miR-1179 were as follows: 95°C for 20 s, followed by 40 cycles of 94°C for 15 s, and 57°C for 1 min. LINC01929 expression was normalized to that of glyceraldehyde 3-phosphate dehydrogenase (GAPDH), and miR-1179 expression to RNU6-2 by using the 2^−ΔΔCt^ method.

### Dual-Luciferase Reporter Assay

Cells were seeded onto 96-well plates, cultured for 24 h, and then transfected with miR-1179 inhibitor or the negative control. Twenty-four hours after transfection, the cells were co-transfected with constructed wild-type or mutated LINC01929 reporter plasmids (WT-LINC01929 or MUT-LINC01929). Forty-eight hours after co-transfection, the luciferase reporter assay was conducted at a Dual-Luciferase Reporter Assay System (Promega, United States). Luminescence intensities of firefly luciferase were finally normalized to those of Renilla luciferase.

### XTT Proliferation Assays

Cell proliferation was determined by XTT Cell Proliferation Assay Kit (AmyJet, China) as per the manufacturer’s instruction [18]. Briefly, suspensions of 2.5 × 10^3^ transfected NCI-H522 or SK-MES-1 cells were seeded in 100 μL of RPMI-1640 medium (HyClone, United States) in 96-well plates. At 0, 24, 48, and 72 h of incubation at 37°C, 50 μL of prepared XTT working solution was added to the well. After another 2 h of incubation, the absorbance was read at 490 nm using a Multiskan MK3 (Thermo Fisher Scientific, United States).

### Transwell Migration and Invasion Assays

Transwell chambers (Corning, United States) were purchased for migration assay, whereas coated Transwell chambers with Matrigel Matrix (Corning) were prepared for invasion assay [19]. The upper inserts were seeded with 4×10^4^ transfected NCI-H522 and SK-MES-1 cells in serum-free RPMI-1640 medium. The lower chambers were filled with 10% FBS-RPMI-1640 medium. After 24 h incubation for migration or 36 h incubation for invasion, the cells that passed the membrane were fixed and stained with 0.2% crystal violet. Images were taken by Leica microsystems (Leica DMi8 Inverted Microscope, GE) and the cells were counted.

### Statistical Analysis

Data were summarized as means ± SD. *p* < 0.05 was served as statistical significance. Student’s t-test, one-way or two-way ANOVA was performed for comparison among two, three, or more groups. The evaluation of the correlation between LINC01929 and miR-1179 expression was analyzed by Pearson’s correlation test. The association of LINC01929 expression and key clinical parameters was assessed using chi-square tests. Kaplan-Meier curves indicating the event and the final times were constructed to evaluate patient overall survival, with comparison analyzed by the log-rank test. Univariate and multivariate COX were used to estimate the significance of clinical characteristics to overall survival.

## Results

### Expression of LINC01929 in NSCLC

**FIGURE 1:**
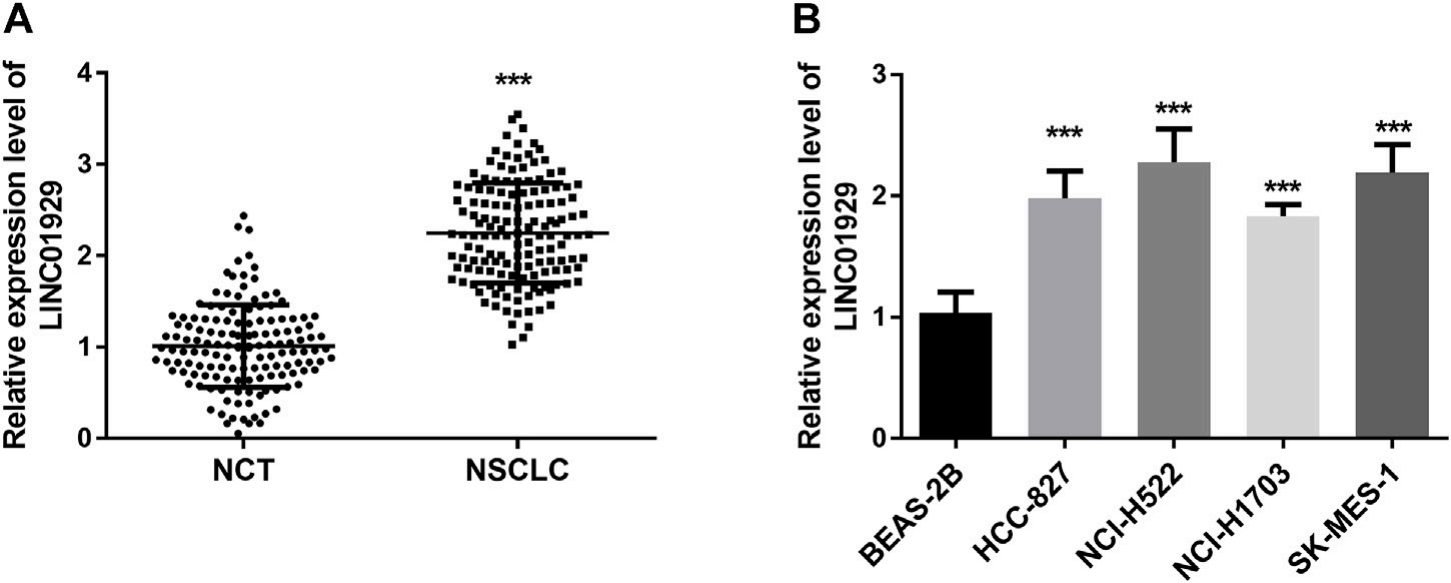
RT-qPCR analysis of LINC01929 expression in NSCLC tissues and cells. **(A)** LINC01929 level in NSCLC tissues is upregulated in comparison with non-cancerous tissues (NCT); **(B)** Expression levels of LINC01929 in NSCLC cell lines (A549, HCC827, NCI-H441, and NCI-H1734) were higher than the level in normal human lung cell line BEAS-2B. The relative expression of LINC01929 was normalized with GAPDH and presented as the mean ± SD. ***p < 0.001.

To determine the expression level of LINC01929 in NSCLC, LINC01929 expression patterns were detected by RT-qPCR and compared in NSCLC tissues and adjacent non-cancerous tissues. An upregulated expression of LINC01929 was observed in NSCLC tissues when compared with adjacent healthy tissues (*p* < 0.001, Figure 1A). LINC01929 differential expression in cancerous and non-cancerous lung tissue was further corroborated by the expression level in cell lines. The results of the RT-qPCR, showed LINC01929 elevated expression in the cell lines of NSCLC (HCC-827, NCI-H522, NCI-H1703, and SK-MES-1) compared with the non-cancerous cell line BEAS-2B (*p* < 0.001, Figure 1B).

### Elevated LINC01929 Expression was Correlated With Clinical Parameters

To examine the potential association between LINC1929 expression and clinical parameters, the chi-square test was conducted. The median value of LINC01929 expression level in 143 NSCLC tissues served as a cut-off to divide the patients into a high-level and low-level group. It was observed that NSCLC samples with high LINC01929 expression were correlated with advanced clinical T stage (*p* = 0.002) and clinical N stage (*p* = 0.010) (Table 1). These data indicate that LINC01929 may be involved in the progression of NSCLC.

### Elevated LINC00473 Expression was Associated With Poor Prognosis in NSCLC

**FIGURE 2:**
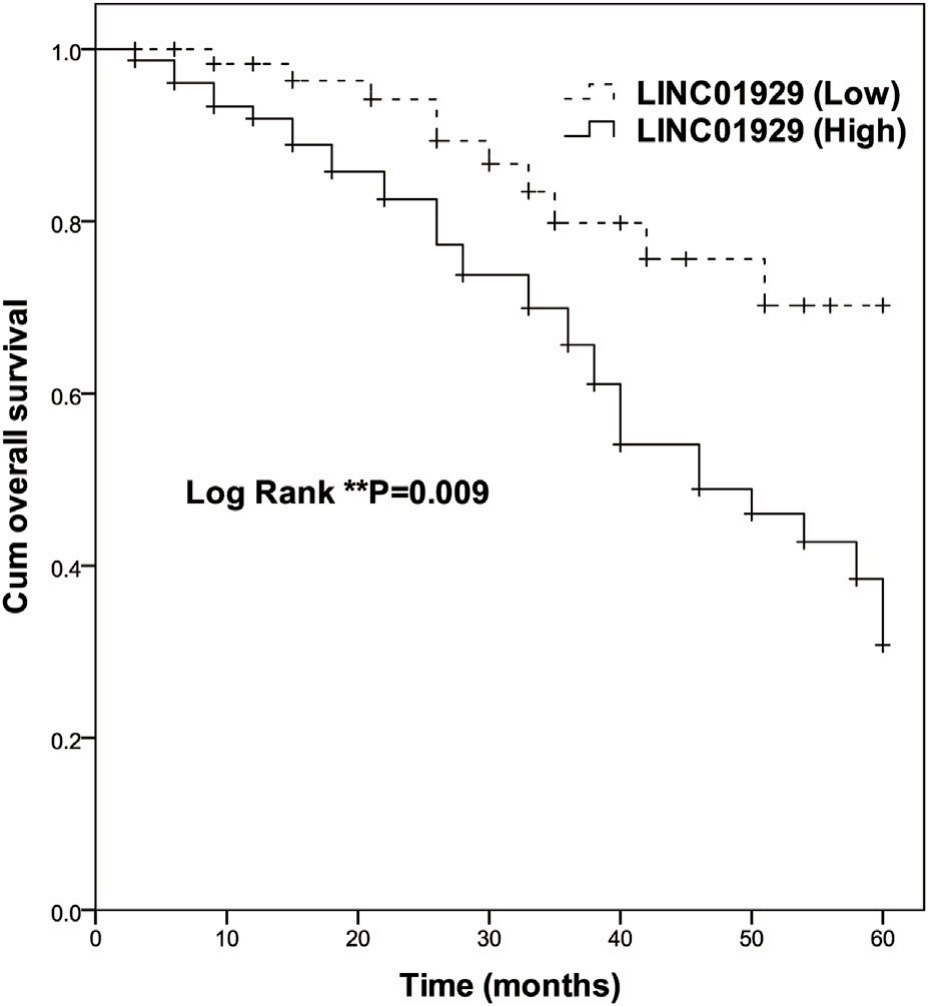
Kaplan-Meier survival analysis of high LINC01929 expression (*n* = 65) and low LINC01929 expression (*n* = 78) in NSCLC patients (Log-rank *p* = 0.009). Enhanced LINC01929 expression is significantly correlated with poor survival.

To investigate the prognostic significance of LINC01929 expression in NSCLC, Kaplan-Meier and Cox analyses were performed. The Kaplan-Meier curve showed a highly significant difference in overall survival of patients with high expression (*n* = 78) and low expression (*n* = 65) groups (*p* = 0.009, Figure 2). At the end of follow-up, only ten in the low LINC01929 expression group had died, compared to 32 in the high LINC01929 expression group. Table 2 shows the significance of various factors correlated with the prognosis of NSCLC using univariate and multivariate Cox analysis. Compared with other factors, high LINC01929 expression was significantly associated with poor survival in univariate Cox analysis (HR: 2.485, 95%CI: 1.220–5.060, *p* = 0.012) and remained significant after multivariate Cox analysis (HR: 3.021, 95%CI: 1.377–6.628, *p* = 0.006). Collectively, these data support the hypothesis that LINC01929 is a promising prognostic factor for NSCLC.

**TABLE 2:** Mortality risk based on independent variables associated with overall survival.

Study variables	Univariate analysis			Multivariate analysis		
	HRs	95% CIs	*p*	HRs	95% CIs	*p*
Age	1.124	0.613–2.062	0.705	1.354	0.669–2.743	0.400
Gender	1.076	0.585–1.979	0.814	1.014	0.540–1.904	0.967
Tumor size	1.540	0.838–2.831	0.164	1.516	0.750–3.063	0.247
Smoking status	1.222	0.666–2.243	0.517	1.602	0.832–3.085	0.159
Pathological type	1.052	0.562–1.967	0.874	1.601	0.810–3.164	0.175
Clinical T stage	2.027	1.105–3.717	0.022	1.961	1.034–3.718	0.039
Clinical N stage	1.382	0.724–2.637	0.326	1.977	1.006–3.887	0.048
Differentiation	1.181	0.632–2.205	0.602	1.651	0.846–3.222	0.141
LINC01929	2.485	1.220–5.060	0.012	3.021	1.377–6.628	0.006

### LINC01929 Functions as a Sponge of miR-1179

**FIGURE 3:**
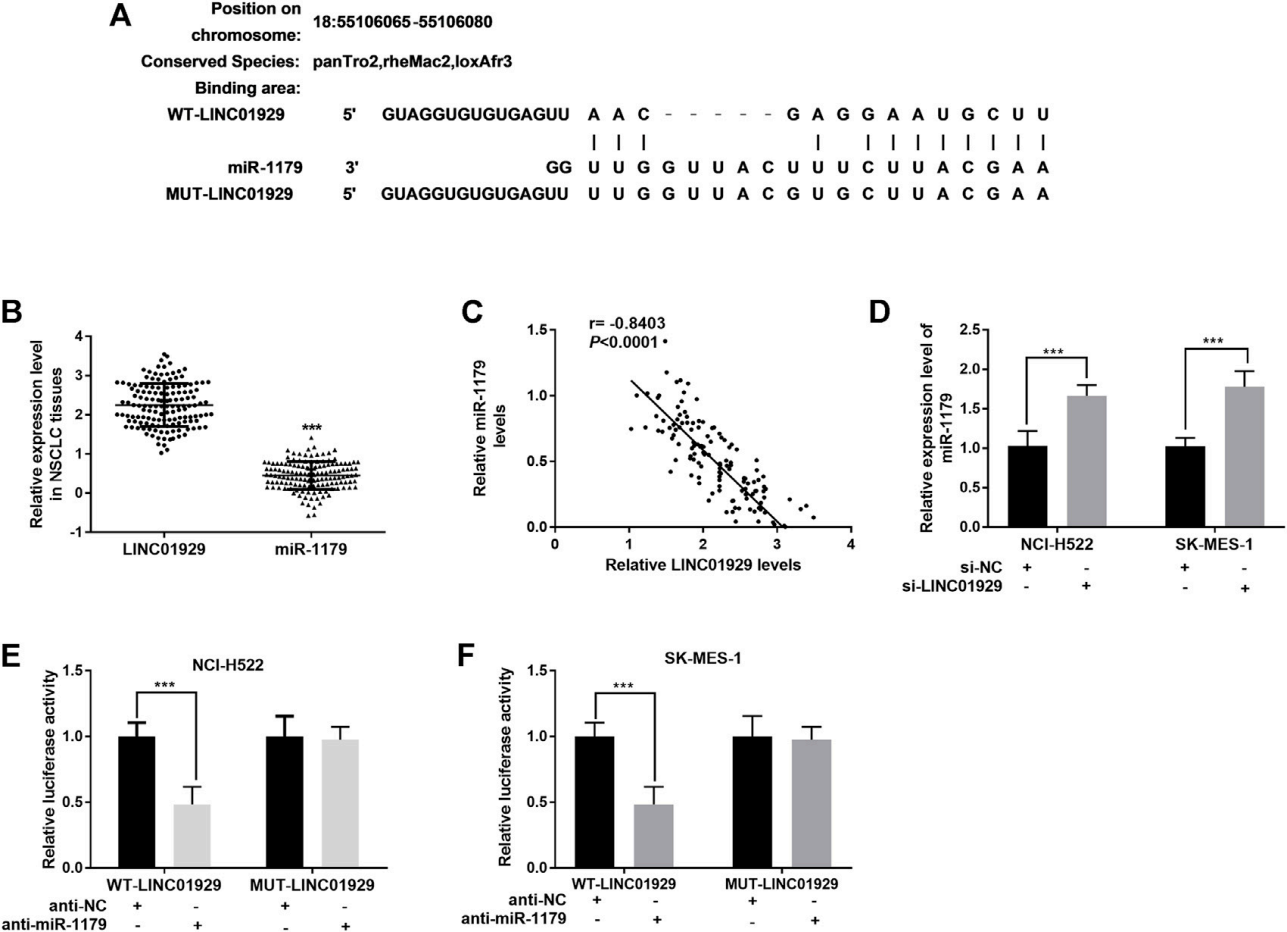
LINC01929 acts as ceRNA of miR-1179. **(A)** Potential binding sites for LINC01929 on miR-1179. **(B)** Comparison of the LINC01929 expression and miR-1179 expression in NSCLC tissues detected by RT-PCR. **(C)** Negative correlation between LINC01929 and miR-1179 expression. **(D)** LINC01929 reversely regulates the expression of miR-1179 in NSCLC cells. “+” indicated “presence”, “−” indicated “absence”. **(E,F)** Dual-luciferase reporter assay performed in NSCLC cells. “+” indicated “presence”, “−” indicated “absence”. **p < 0.01, ***p < 0.001.

To investigate LINC01929-interacting miRNA, an analysis was carried out on LncBase Predicted v.2 (http://carolina.imis.athena-innovation.gr/diana_tools/web/). miR-1179, a decreased miRNA in lung cancer[20], was found to be a target of LINC01929 (Figure 3A). The miR-1179 relative expression levels in NSCLC tissues were also detected as lower than the LINC01929 relative expression levels. (*p* < 0.001, Figure 3B). and inversely correlated with LINC01929 expression (r = −0.8403, *p* < 0.001; Figure 3C). Furthermore, the inhibition of LINC01929 resulted in an increase of miR-1179 expression in NCI-H522 and SK-MES-1 cells (*p* < 0.001, Figure 3D). The data obtained from the dual-luciferase reporter assay directly verified the combination between LINC01929 and miR-1179. miR-1179 suppression elevated the luciferase activity of wide-type LINC01929 containing the predicted binding sites (*p* < 0.001, Figures 3E,F). All these data support miR-1179 is a LINC01929-associated miRNA and LINC01929 may act as a sponge of miR-1179.

### *In Vitro* Approaches Revealed Functions of LINC01929 in the NSCLC Cells in Part Through Interacting With miR-1179

**FIGURE 4:**
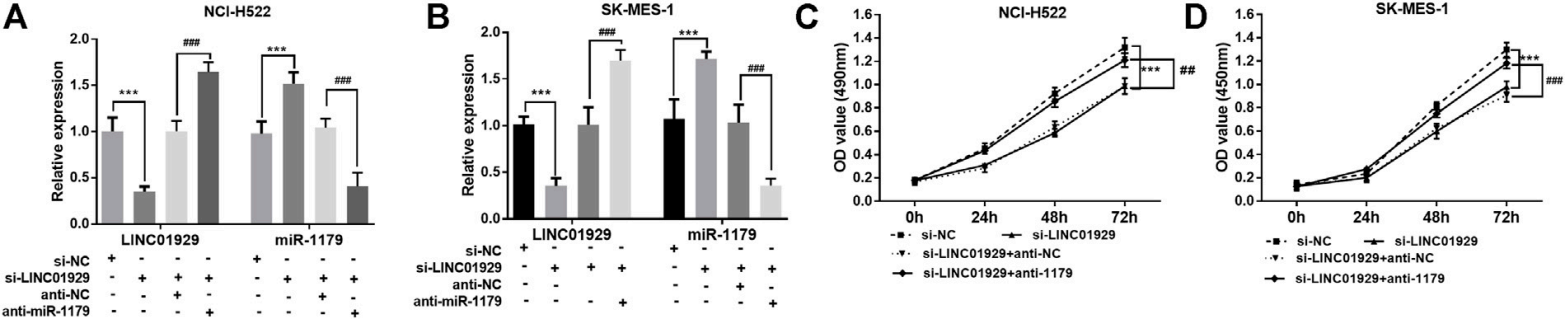
LINC01929 knockdown inhibits NSCLC cell proliferation via miR-1179. **(A,B)** RT-qPCR analysis of the RNA interference efficiency. SK-MES-1 and NCI-H522 cells were transfected with si -LINC01929 or co-transfected with si -LINC01929 and anti-miR-1179. The expressions of LINC01929 and miR-1179 were then examined. **(C,D)** XTT assays showing the proliferation of SK-MES-1 and NCI-H522 cells transfected with si -LINC01929 or co-transfected with si -LINC01929 and anti-miR-1179. Data are presented as the mean ± SD. “+” indicated “presence”, “−” indicated “absence”. ***p < 0.001, vs. si-NC; ^##^ *p* < 0.01, ^###^ *p* < 0.001, vs. si-LINC01929+anti-miR-1179.

**FIGURE 5:**
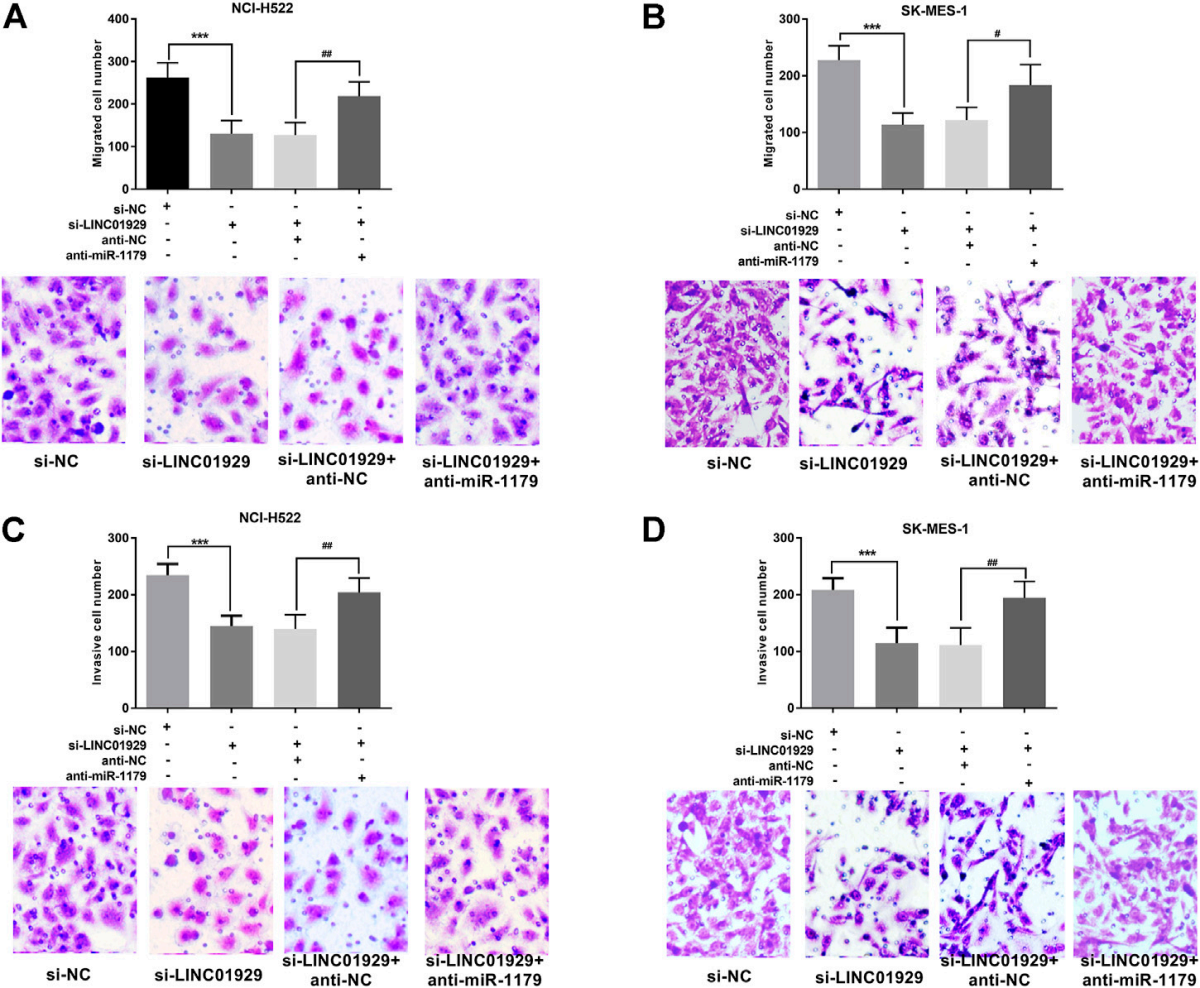
LINC01929 knockdown inhibits NSCLC cell invasion and migration via miR-1179. **(A,B)**The motility of SK-MES-1 and NCI-H522 cells after transfection assessed by Transwell assay, along with the representative pictures. LINC01929 inhibition decreased the migratory cells, whereas miR-1179 inhibitor can restore the cell migratory ability partly. **(C,D)** Invasive ability SK-MES-1 and NCI-H522 cells after transfection measured by modified transwell assay, along with the representative pictures. LINC01929 knockdown decreased the invasive cells, whereas miR-1179 inhibitor can recover the cell invasive capability partly. “+” indicated “presence”, “−” indicated “absence”. ***p < 0.001, vs. si-NC; ^#^ *p* < 0.05, ^##^ *p* < 0.01, vs. si-LINC01929+anti-miR-1179.

Next, the functional significance of LINC01929 dysregulation was investigated in LINC01929 specific siRNA transfected NCI-H522 and SK-MES-1 cells. The transfection efficiency in cells was verified by RT-qPCR (*p* < 0.001, Figures 4A,B). Then, we explored the functional impact of LINC01929 knockdown on cell proliferation using an XTT assay. It was observed that LINC01929 si-RNA caused a reduction in cell proliferation of NCI-H522 and SK-MES-1. Nevertheless, these effects were reversed by the co-transfection with miR-1179 inhibitor (*p* < 0.001, Figures 4C,D). The transwell assay showed the functional significance of LINC01929 in cell migration and invasion. LINC01929 knockdown reduced migrated cells in NCI-H522 and SK-MES-1 cells, but the employ of miR-1179 inhibitor recovered cell migration (*p* < 0.05, Figures 5A,B). Similar effects of single LINC1929 inhibition or LINC1929 and miR-1179 co-inhibition on the NSCLC cell invasion were also observed (*p* < 0.01, Figures 5C,D). These data demonstrate that LINC01929 promotes lung cancer cell growth, migration, and invasion partly by moderating the expression of miR-1179.

## Discussion

As is known, lncRNAs participate in a series of different cellular processes, such as chromatin remodeling and RNA stability [21]. The existing classifications for lncRNAs rest on their properties, from their size or their localization to their function [22]. For instance, long intergenic non-coding RNAs (lincRNAs) are defined as autonomously transcribed non-coding RNAs containing more than 200 nucleotides and with no overlap with annotated coding genes [23]. LincRNAs possess the same features as the other transcripts of the lncRNAs family and comprise more than half of lncRNAs in humans [24]. They often exhibit tissue specificity and may moderate the expression of their target genes in a tissue-specific manner [24]. Recent studies raised the possibility that this tissue specificity may reflect the potential of lncRNAs as biomarkers for lung cancer. For instance, downregulation of LINC00460 inhibited the growth and promoted the apoptosis of pancreatic cancer cells, showing potential as a novel biomarker and promising therapeutic strategy for pancreatic cancer [25].

In this study, the expression level of LINC01929 was detected by RT-qPCR, and elevated levels were observed in NSCLC tissues and cells in comparison with normal ones. Highly expressed LINC01929 has also been observed in oral squamous cell carcinoma tissue samples and cell lines [26, 27]. Previous research has verified that upregulation of LINC01929 can be used to predict the overall survival of hepatocellular carcinoma patients without fibrosis [28]. In our study, the increased expression was analyzed to be associated with advanced clinical T and N stages. These findings imply that LINC01929 is involved in the progression of NSCLC and may be a useful prognostic biomarker for NSCLC. The overall survival of patients in our study was plotted as a Kaplan-Meier curve, to show the discrimination ability of LINC01929 in prognosis prediction. Further, the potential of LINC01929 as an independent prognostic factor was verified by multi-variate COX analysis. In agreement with these findings, the upregulation of LINC01929 has previously been screened as a lncRNA associated with survival in non-small cell lung carcinoma patients [29]. Collectively, the data supports the promising potential for LINC01929 as a prognostic factor in NSCLC.

When focusing on lncRNA molecular mechanisms, it has been proposed that they may act to sponge miRNAs and regulate mRNA activity by competing for endogenous RNAs (ceRNAs) [30]. Any lincRNA that shares a sequence with another RNA (coding or non-coding) could function as an RNA decoy and potentially be a ceRNA. There is emerging evidence that lincRNAs may be directly implicated in human disease pathogenesis [31]. Previously, LINC01929 was identified as existing in the cytoplasm through a nuclear-cytoplasmic fractionation assay and that it may sponge miR-137-3p in oral squamous cell carcinoma cells [26]. Based on the fact that lincRNAs have roughly similar nuclear-cytoplasmic enrichment ratios across cell types [32], we deduced LINC01929 may also act as a ceRNA in NSCLC cells and play its role in cellular function by moderating miRNA expression. To verify this, the binding sites between LINC01929 and miR-1179 were predicted. Moreover, the expression of miR-1179 was inversely correlated with LINC01929 expression, and inhibition of LINC01929 lead to an increase in miR-1179 levels. The dual-luciferase reporter assay directly verified the interaction between LINC01929 and miR-1179. Similarly, miR-1179 has been verified to be sponged by LINC00857 in lung adenocarcinoma cells [33]. Thus, miR-1179 may be a LINC01929-target miRNA.

As reported previously, LINC01929 functioned as a tumor-promoting molecule in oral squamous cell carcinoma by accelerating cell proliferation, migration, and invasion, and suppressing apoptosis, suggesting that it may be a novel target for cancer therapy [26]. With regards to the relationship of LINC01929 and miR-1179, a cellular function analysis was performed by LINC01929 knockdown or both LINC01929 and miR-1179 knockdowns. Utilizing XTT proliferation assays and Transwell migration and invasion assays, inhibition of LINC01929 caused declines in cell proliferation, migration, and invasion. However, these effects were reversed by co-downregulation of LINC01929 and miR-1179. As highlighted previously, miR-1179 inhibits the growth and invasion of NSCLC cells by targeting sperm-associated antigen 5 (SPAG5) [20]. SPAG5 is an emerging oncogene in lung cancer, and associated with unfavorable prognosis in patients with lung adenocarcinoma [34]. In addition, the links between lncRNAs and miRNAs can influence specific gene expression [30]. In NSCLC, LINC01929 may promote cancer progression in part through sponging miR-1179 and this may then influence SPAG5.

## Conclusion

In summary, this study found that LINC01929 was up-regulated in NSCLC tissues and cell lines and was associated with later clinical stage of the disease. Furthermore, downregulation of LINC01929 inhibited cellular proliferation, migration, and invasion. The study provides support for LINC01929 as a novel prognostic biomarker for NSCLC.

## Summary Table

### What is Known About This Subject

• Non-small cell lung carcinoma constitutes most lung cancer and may lead to a poor prognosis.
• LncRNAs are implicated in lung cancer progression and have the potential as biomarkers of prognosis.
• LINC01929 was screened as a dysregulated lncRNA in NSCLC.

### What This Paper Adds

• LINC01929 was up regulated in NSCLC tissues and cells.
• Inhibition of LINC01929 reduced the progression of NSCLC.
• miR-1179 was a target miRNA of LINC01929.
• LINC01929 was a promising prognostic factor and therapy target for NSCLC.

## Summary Sentence

This work represents an advance in biomedical science because our study provides support for LINC01929 as a novel prognostic biomarker and therapy target for NSCLC.

## Data Availability

The original contributions presented in the study are included in the article/supplementary material, further inquiries can be directed to the corresponding author.

## References

[1] Siegel RL Miller KD Jemal A. Cancer Statistics, 2020. CA Cancer J Clin (2020) 70(1):7–30. 10.3322/caac.21590 31912902

[2] Bade BC Dela Cruz CS. Lung Cancer 2020: Epidemiology, Etiology, and Prevention. Clin Chest Med (2020) 41(1):1–24. 10.1016/j.ccm.2019.10.001 32008623

[3] Nasim F Sabath BF Eapen GA. Lung Cancer. Med Clin North Am (2019) 103(3):463–73. 10.1016/j.mcna.2018.12.006 30955514

[4] Yang D Liu Y Bai C Wang X Powell CA. Epidemiology of Lung Cancer and Lung Cancer Screening Programs in China and the United States. Cancer Lett (2020) 468:82–7. 10.1016/j.canlet.2019.10.009 31600530

[5] Duma N Santana-Davila R Molina JR. Non-Small Cell Lung Cancer: Epidemiology, Screening, Diagnosis, and Treatment. Mayo Clin Proc (2019) 94(8):1623–40. 10.1016/j.mayocp.2019.01.013 31378236

[6] Chi A Fang W Sun Y Wen S. Comparison of Long-Term Survival of Patients with Early-Stage Non-small Cell Lung Cancer after Surgery vs Stereotactic Body Radiotherapy. JAMA Netw Open (2019) 2(11):e1915724. 10.1001/jamanetworkopen.2019.15724 31747032 PMC6902813

[7] Yang CJ Gu L Shah SA Yerokun BA D'Amico TA Hartwig MG Long-term Outcomes of Surgical Resection for Stage IV Non-small-cell Lung Cancer: A National Analysis. Lung Cancer (Amsterdam, Netherlands) (2018) 115:75–83. 10.1016/j.lungcan.2017.11.021 29290266

[8] de Sousa VML Carvalho L. Heterogeneity in Lung Cancer. Pathobiology (2018) 85(1-2):96–107. 10.1159/000487440 29635240

[9] Zito Marino F Bianco R Accardo M Ronchi A Cozzolino I Morgillo F Molecular Heterogeneity in Lung Cancer: From Mechanisms of Origin to Clinical Implications. Int J Med Sci (2019) 16(7):981–9. 10.7150/ijms.34739 31341411 PMC6643125

[10] Terashima M Ishimura A Wanna-Udom S Suzuki T. MEG8 Long Noncoding RNA Contributes to Epigenetic Progression of the Epithelial-Mesenchymal Transition of Lung and Pancreatic Cancer Cells. J Biol Chem (2018) 293(47):18016–30. 10.1074/jbc.RA118.004006 30262664 PMC6254362

[11] Mari-Alexandre J Diaz-Lagares A Villalba M Juan O Crujeiras AB Calvo A Translating Cancer Epigenomics into the Clinic: Focus on Lung Cancer. Transl Res (2017) 189:76–92. 10.1016/j.trsl.2017.05.008 28644958

[12] Tao H Yang JJ Zhou X Deng ZY Shi KH Li J Emerging Role of Long Noncoding RNAs in Lung Cancer: Current Status and Future Prospects. Respir Med (2016) 110:12–9. 10.1016/j.rmed.2015.10.006 26603340

[13] Chen Z Lei T Chen X Gu J Huang J Lu B Long Non-coding RNA in Lung Cancer. Clin Chim Acta (2020) 504:190–200. 10.1016/j.cca.2019.11.031 31790697

[14] Ren MM Xu S Wei YB Yang JJ Yang YN Sun SS Roles of HOTAIR in Lung Cancer Susceptibility and Prognosis. Mol Genet Genomic Med (2020) 8(7):e1299. 10.1002/mgg3.1299 32394637 PMC7336741

[15] Yu X Zhang Y. Identification of a Long Non-coding RNA Signature for Predicting Prognosis and Biomarkers in Lung Adenocarcinoma. Oncol Lett (2020) 19(4):2793–800. 10.3892/ol.2020.11400 32218832 PMC7068299

[16] Ding J Cao J Chen Z He Z. The Role of Long Intergenic Noncoding RNA 00511 in Malignant Tumors: A Meta-Analysis, Database Validation and Review. Bioengineered (2020) 11(1):812–23. 10.1080/21655979.2020.1795384 32713253 PMC8291795

[17] Rusch VW Asamura H Watanabe H Giroux DJ Rami-Porta R Goldstraw P. The IASLC Lung Cancer Staging Project: A Proposal for a New International Lymph Node Map in the Forthcoming Seventh Edition of the TNM Classification for Lung Cancer. J Thorac Oncol (2009) 4(5):568–77. 10.1097/JTO.0b013e3181a0d82e 19357537

[18] Sizdahkhani S Feldman MJ Piazza MG Ksendzovsky A Edwards NA Ray-Chaudhury A Somatostatin Receptor Expression on Von Hippel-Lindau-Associated Hemangioblastomas Offers Novel Therapeutic Target. Sci Rep (2017) 7:40822. 10.1038/srep40822 28094316 PMC5240113

[19] Lu X Chen L Li Y Huang R Meng X Sun F Long Non-coding RNA LINC01207 Promotes Cell Proliferation and Migration but Suppresses Apoptosis and Autophagy in Oral Squamous Cell Carcinoma by the microRNA-1301-3p/lactate Dehydrogenase Isoform A axis. Bioengineered (2021) 12:7780–93. 10.1080/21655979.2021.1972784 34463208 PMC8806684

[20] Song L Dai Z Zhang S Zhang H Liu C Ma X MicroRNA-1179 Suppresses Cell Growth and Invasion by Targeting Sperm-Associated Antigen 5-mediated Akt Signaling in Human Non-small Cell Lung Cancer. Biochem Biophys Res Commun (2018) 504(1):164–70. 10.1016/j.bbrc.2018.08.149 30180955

[21] Tsagakis I Douka K Birds I Aspden JL. Long Non-coding RNAs in Development and Disease: Conservation to Mechanisms. J Pathol (2020) 250(5):480–95. 10.1002/path.5405 32100288 PMC8638664

[22] Ma L Bajic VB Zhang Z. On the Classification of Long Non-coding RNAs. RNA Biol (2013) 10(6):925–33. 10.4161/rna.24604 23696037 PMC4111732

[23] St Laurent G Wahlestedt C Kapranov P. The Landscape of Long Noncoding RNA Classification. Trends Genet (2015) 31(5):239–51. 10.1016/j.tig.2015.03.007 25869999 PMC4417002

[24] Ransohoff JD Wei Y Khavari PA. The Functions and Unique Features of Long Intergenic Non-coding RNA. Nat Rev Mol Cel Biol (2018) 19(3):143–57. 10.1038/nrm.2017.104 PMC5889127 29138516

[25] Cheng J Lou Y Jiang K. Downregulation of Long Non-coding RNA LINC00460 Inhibits the Proliferation, Migration and Invasion, and Promotes Apoptosis of Pancreatic Cancer Cells via Modulation of the miR-320b/ARF1 axis. Bioengineered (2021) 12(1):96–107. 10.1080/21655979.2020.1863035 33345740 PMC8806231

[26] Che H Che Y Zhang Z Lu Q. Long Non-coding RNA LINC01929 Accelerates Progression of Oral Squamous Cell Carcinoma by Targeting the miR-137-3p/FOXC1 Axis. Front Oncol (2021) 11:657876. 10.3389/fonc.2021.657876 33968763 PMC8097103

[27] Liu W Gan CY Wang W Liao LD Li CQ Xu LY Identification of lncRNA-Associated Differential Subnetworks in Oesophageal Squamous Cell Carcinoma by Differential Co-expression Analysis. J Cel Mol Med (2020) 24(8):4804–18. 10.1111/jcmm.15159 PMC7176870 32164040

[28] Ye J Wu S Pan S Huang J Ge L. Risk Scoring Based on Expression of Long Non-coding RNAs Can Effectively Predict Survival in Hepatocellular Carcinoma Patients with or without Fibrosis. Oncol Rep (2020) 43(5):1451–66. 10.3892/or.2020.7528 32323856 PMC7108035

[29] Acha-Sagredo A Uko B Pantazi P Bediaga NG Moschandrea C Rainbow L Long Non-coding RNA Dysregulation is a Frequent Event in Non-small Cell Lung Carcinoma Pathogenesis. Br J Cancer (2020) 122(7):1050–8. 10.1038/s41416-020-0742-9 32020063 PMC7109049

[30] Chan JJ Tay Y. Noncoding RNA:RNA Regulatory Networks in Cancer. Int J Mol Sci (2018) 19:E1310. 10.3390/ijms19051310 29702599 PMC5983611

[31] Zhang H Liu S Tang L Ge J Lu X. Long Non-coding RNA (LncRNA) MRPL23-AS1 Promotes Tumor Progression and Carcinogenesis in Osteosarcoma by Activating Wnt/β-Catenin Signaling via Inhibiting microRNA miR-30b and Upregulating Myosin Heavy Chain 9 (MYH9). Bioengineered (2021) 12(1):162–71. 10.1080/21655979.2020.1863014 33356805 PMC8806232

[32] Cabili MN Trapnell C Goff L Koziol M Tazon-Vega B Regev A Integrative Annotation of Human Large Intergenic Noncoding RNAs Reveals Global Properties and Specific Subclasses. Genes Dev (2011) 25(18):1915–27. 10.1101/gad.17446611 21890647 PMC3185964

[33] Wang L Cao L Wen C Li J Yu G Liu C LncRNA LINC00857 Regulates Lung Adenocarcinoma Progression, Apoptosis and Glycolysis by Targeting miR-1179/SPAG5 axis. Hum Cel (2020) 33(1):195–204. 10.1007/s13577-019-00296-8 31667785

[34] Huang R Li A. SPAG5 Is Associated with Unfavorable Prognosis in Patients with Lung Adenocarcinoma and Promotes Proliferation, Motility and Autophagy in A549 Cells. Exp Ther Med (2020) 20(5):77. 10.3892/etm.2020.9205 32968434 PMC7500011

